# Genome sequences of *Microbacterium foliorum* phages, BabyYoda and Lynlen

**DOI:** 10.1128/mra.01006-23

**Published:** 2023-12-22

**Authors:** Ashley E. Prout, Sarah J. Swerdlow

**Affiliations:** 1 Biology Department, Thiel College, Greenville, Pennsylvania, USA; 2 Department of Natural Sciences-Biological Sciences, University of Pittsburgh-Greensburg, Greensburg, Pennsylvania, USA; Loyola University Chicago, Chicago, Illinois, USA

**Keywords:** bacteriophages, genome analysis

## Abstract

BabyYoda and Lynlen are two cluster EB phages that were discovered at Thiel College using *Microbacterium foliorum* NRRL B-24224. Both BabyYoda and Lynlen are predicted to be lytic, with siphovirus morphologies, with genome sizes of 41,557 and 41,448 base pairs, respectively.

## ANNOUNCEMENT

Bacteriophages are the most abundant organisms on Earth. The discovery and genome analyses for a broad diversity of bacteriophages can reveal their evolutionary history and population structures and facilitate the development of bacteriophages as therapeutics for treating bacterial infections (1, 2). Here, we present the isolation and genomic analyses of two phages isolated using *Microbacterium foliorum* NRRL B-24224 phages, Lynlen and BabyYoda, discovered in 2018 and 2020, respectively.

Both phages were isolated from soil samples collected in Pennsylvania, USA (Table 1), by suspending ~1.5 g of soil in 30 mL of peptone-yeast extract-calcium chloride (PYCa) media and shaking at 225 rpm at 30°C for 2 hours (3). The suspension was then spun at 1,200 rpm for 10 minutes, followed by filtration through a 0.22 µM syringe filter (3). The filtrate was either plated directly in PYCa top agar with *M. foliorum,* yielding plaques of phage Lynlen, or inoculated with *M. foliorum* and incubated at 30°C with shaking for 48 hours (3) prior to plating, yielding plaques of phage BabyYoda. Both phages were purified through three rounds of plating.

**TABLE 1:** Genome, sample, and plaque characteristics of Lynlen and BabyYoda bacteriophages

Phage	Lynlen	BabyYoda
Year found	2018	2020
Enriched sample^*a*^	No	Yes
Plaque diameter (mm)	1	3
GPS coordinates	41.4125 N, 80.3883 W	41.4142 N, 80.3813 W
Genome accession no.	MT310896	MW601221
SRA accession no.	SRX21748120	SRX21748106
Genome ends	3′ sticky overhang	3′ sticky overhang
Genome size (bp)	41448	41557
GC percentage (%)	66.9	68.7
Coverage	3,005X	12,000X
No. of genes	72	69
No. of genes with putative functional assignments (%)	36.1	39.1

^*a*^ Enriched sample: inoculated with *Microbacterium foliorum* and incubated at 30°C with shaking for 48 hours (3) prior to plating.

DNA was isolated from a lysate of each phage using the Promega Wizard DNA cleanup kit, prepared for sequencing using NEB Ultra II Library Kit, and sequenced using an Illumina MiSeq (v3 reagents), yielding 870,466 150-base single-end reads for Lynlen and 1,892,506 300-base paired reads for BabyYoda. Raw reads were assembled using Newbler v2.9 and checked for accuracy, coverage, and genomic termini using Consed v29 (4). The genome length, genome ends, and G + C content for Lynlen and BabyYoda can be found in Table 1. Both phages were assigned to the EB cluster based on a minimum of 35% gene content similarity between the phages in the Actinobacteriophage Database, PhagesDB (5, 6).

Both genomes were annotated utilizing DNA Master (https://cobamide2.bio.pitt.edu), GLIMMER (7), GeneMark v1.2 (8), Phamerator (9), Startorator (http://phages.wustl.edu/starterator/), and PECAAN (https://discover.kbrinsgd.org/). tRNA genes were analyzed utilizing Aragorn (10) and tRNAscan-SE v2.0 (11), revealing tRNAs for asparagine (Asn) and glutamine encoded by BabyYoda and Asn and lysine (Lys) for Lynlen. Putative gene functions were assigned based on sequencing homology using BLASTp (12) (actinobacteriophage and nonredundant protein databases) and HHPRED (13) (PDB mmCIF70, Pfam-A, and NCBI Conserved Domain databases). Default parameters were used for all programs. In Table 1, the number of genes and the percentage of genes with a putative function are indicated.

Both phages transcribe most genes in the forward direction with two to three genes transcribed in reverse. Lynlen and BabyYoda contain a ParB-like nuclease domain (14) (gp50 and gp49, respectively) and an endolysin gene (15), which are consistent among EB phages (5). However, the endolysin gene (gp22) in Lynlen is 48 bp shorter than in BabyYoda (gp21) (5). The different endolysin gene sizes are present among additional clusters including cluster E phages (5, 13). Interestingly, BabyYoda (gp32) contains a HicA-like toxin gene which is shared among many clusters including EB, CR, DO, and DR; however, Lynlen does not contain this gene (5, 16).

## Data Availability

BabyYoda is available at GenBank with accession no. MW601221 and Sequence Read Archive (SRA) no. SRX21748106. Lynlen is available at GenBank with accession no. MT310896 and Sequence Read Archive (SRA) no. SRX21748120.
